# Undergraduate Medical Students’ Search for Health Information Online: Explanatory Cross-Sectional Study

**DOI:** 10.2196/16279

**Published:** 2020-03-02

**Authors:** Teresa Loda, Rebecca Erschens, Florian Junne, Andreas Stengel, Stephan Zipfel, Anne Herrmann-Werner

**Affiliations:** 1 Medical Department VI/Psychosomatic Medicine and Psychotherapy University Hospital Tuebingen Tuebingen Germany; 2 Charité Center for Internal Medicine and Dermatology Department for Psychosomatic Medicine Charité-Universitätsmedizin Berlin Berlin Germany; 3 Corporate Member of Freie Universität Berlin Humboldt-Universität zu Berlin and Berlin Institute of Health Berlin Germany; 4 Deanery of Students’ Affairs Faculty of Medicine University Hospital Tuebingen Tuebingen Germany

**Keywords:** digital health literacy, medical education, evidence-based online information, digital native, trustworthy webpages

## Abstract

**Background:**

Previous research shows that being a “digital native” or growing up in a digital environment does not necessarily lead to increased digital competencies, such as digital health literacy or evaluation of webpage quality.

**Objective:**

This study showed how medical students searched for health information online, specifically the recommended testing for histamine intolerance, by comparing the use of various search engines (Google, Medisuch, and a website of the student’s choice) to find out more about search strategies in future health professionals. As Medisuch presents a qualitatively better search engine, we assumed that medical students using this search engine might find valid information faster on more reliable webpages, and might recommend the correct diagnostic steps for histamine intolerance to their patients more often than students using a generic search engine like Google.

**Methods:**

Medical students in their third year of study were asked to find the relevant diagnostic steps of histamine intolerance online. They were randomly assigned to use one search engine: Google, their personal choice, or Medisuch. Their process of seeking information online was video recorded.

**Results:**

In total, 140 medical students participated in this study. The total number of webpages found did not differ among the groups (*P*=.52). Students using Medisuch (*P*=.02) correctly identified the elimination diet as a relevant diagnostic step more frequently. The provocation test was reported by almost half of the students independent of the search engine used. In general, medical students commonly identified trustworthy webpages in all three groups (Google: 36/44, 82%; free choice: 31/36; 86%; and Medisuch: 35/45, 78%).

**Conclusions:**

The results indicate that medical students were able to find trustworthy health-related information online independent of the search engine used. Medical students that are digital natives seem to have proper internet skills and a knowledge of how to use them. They entered specific medical terms (evidence-based diagnostic steps) or names of reliable webpages (DocCheck) in the search engines to gain correct information. However, it remains to be seen if this behavior can be called true “digital literacy”.

## Introduction

The internet is omnipresent and has become the primary source of information for many [1]. It has been reported that 52.2% of Europe’s population has used internet searches for health and health-related issues [2]. Çoklar et al [3] reported that online information search strategies represented one of the most important variables in effective and efficient internet use. Previous research has shown that online search strategies might be influenced and explained by the time spent online, Web experience, and individual difference (like domain knowledge or epistemological belief) [4-7]. An investigation of university students’ online search strategies in different contexts indicated that the students were able to search online for daily life information. However, they had difficulties in the online search strategies used for learning activities, and the authors suggested that teachers should help students develop online search strategies for academic uses [8]. The need for guidance—particularly for people with little expertise—has also been highlighted by Armstrong and Large [9] in their manual on online search strategies.

Furthermore, it has been shown that falling in the category of “digital natives” [10] or showing “digital nativity” [3] (ie, being born in a digital world and, therefore, frequently using internet or media devices since early childhood) does not automatically correlate with online literacy [1,3]. Therefore, attempts have been made to clarify quality indicators for online information.

One such approach involves introducing specific search engines that prefilter for the user and only show reliable results, such as the German webpage Medisuch [11]. Another approach is to certify webpages with trustworthy, evidence-based medical content and introduce certificates that make it easier for average people and professionals to decide quickly if a webpage can be trusted. At first glance, this approach seems easy; however, people seeking information online still have to be properly trained [12]. Examples of certificates available in the topic of health-related information are the afgis certificate and the Health On the Net Foundation Code of Conduct (HONcode) certificate. For a more comprehensive list, see Pauer et al [13].

To properly advise patients, medical students need to be generally aware of diagnostic steps; if they lack the knowledge, they should know how to find reliable information. As medical students are still learning, specific training can impact their attitude and behavior. This study compared various search engines to investigate how medical students search for health information online. The aim of this study was to track medical students’ searches for health information online and identify potentially weak strategies, which could be addressed specifically within the medical curriculum. We wanted to compare three different approaches and focus on the medical students’ findings on histamine intolerance. As Medisuch presents a qualitatively better search engine [11], medical students using it may find valid information faster on more reliable webpages, and may recommend the correct diagnostic steps for histamine intolerance to their patients more often than students using a generic search engine like Google.

## Methods

### Study Design and Participants

This study was performed in an explanatory cross-sectional manner. Third-year students from the Faculty of Medicine of the University Hospital Tuebingen, Germany, were recruited from their curricular courses in the Department of Internal Medicine VI. They had all received theoretical input on functional disorders and differential diagnoses with a focus on intolerances before participating in the study. Group sizes consisted of 8 to 16 students per teaching session. Students were taught by experienced physicians. Participation in the course was mandatory, but participation in the study was on a voluntary basis.

### Ethics

The study received ethical approval from the Ethics Committee of the Tuebingen Medical Faculty (443/2018BO2). All participants gave written informed consent. They did not receive a reimbursement for their participation.

### Process of Study

**Figure 1:**
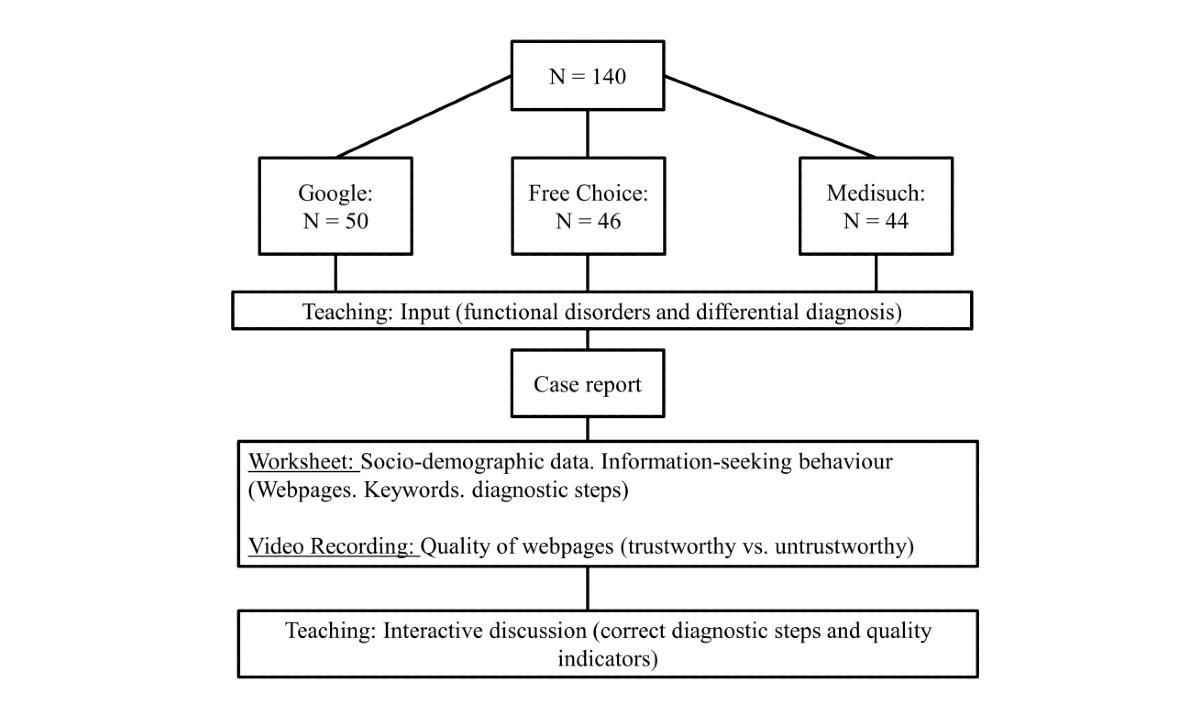
Process of study including teaching and assessment.

Students of the university must attend a 2-week course on psychosomatic medicine in their third year. Every 2 weeks, a new group of approximately 12 to 15 students start the course. The topic is “somatoform disorders” on the third day of the course. Students received a case report involving a patient who consulted her physician due to abdominal pain. The patient in the fictitious case had already researched her health problem beforehand and asked specifically about potential causes such as problems with digestion of histamines. The patient wanted to know which steps had to be taken to rule out or confirm histamine intolerance. Each student had access to one stationary computer with connection to the internet via Internet Explorer or Firefox (both were installed and students were free to choose). Students were instructed to complete a worksheet about histamine intolerance by searching for related information online. They were randomly assigned to one of three groups: Google [14], free choice, or Medisuch [15]. In the free choice group students could choose any search engine they wanted to use. There were no prerequisites for participation, and they were not taught further information about the various search engines. Medisuch is a specific search engine that prefilters the information found and only shows reliable results (ie, medical webpages that are certified or evidence-based) [11]. The students had 10 minutes to research information online and mark their findings on the worksheet, which was collected by a study assistant after the time period. Teaching then continued with an interactive discussion where students were informed about the correct diagnostic steps as well as quality indicators of good webpages and apps. Figure 1 shows an overview of the study. The quality indicators were based on the checklists used by afgis and HONcode, such as objectivity or accuracy. For a more comprehensive list, see Pauer and colleagues [13].

Students were asked about demographic variables such as age and gender, as well as experience with electronics such as possession of electronic devices and time spent online per day. They also rated their competence on the topic of histamine intolerance on a 6-point Likert scale (0 = not competent at all and 5 = highly competent).

### Video Recording

Before starting the teaching session, students were asked to start Morae (TechSmith, Michigan) [16] on their computer, which was used to track students’ online search for health information via a screen recording of their desktop. Morae has been previously applied within the research area of online search behavior by university students [17-20]. The software generates quantitative data on different variables such as time spent on each webpage and clicks made. To the best of our knowledge, no other standardized procedure exists that would successfully analyze the video-recorded data in a qualitative way. In alignment with the previously described procedure [20], we aimed to develop a categorical system (eg, search engine, number of webpages, search strategies) to guarantee standardized analysis and evaluation of the Morae videos.

All Morae videos were coded according to this categorical system by experienced members of the research team. All webpages students had visited were listed alphabetically, and their quality was classified according to a rating sheet by two independent raters. In cases of a disagreement, a third independent rater was consulted.

#### Information-Seeking Behavior

Students answered specific questions about the search engine(s) used, keywords entered, and the number of webpages they consulted before making their diagnostic recommendation. Additionally, they rated the number of pages considered as trustworthy as well as the number of pages they would recommend to a patient. Trustworthy was defined as an evidence-based and certified webpage; thus, untrustworthy was defined as a nonevidence-based one such as an advertisement or self-made webpage by an individual. To get an idea on the effectiveness of medical students’ searches, the number of pages opened and the keywords needed to get to the desired answer were considered as relevant markers.

#### Diagnostic Recommendation

Students had to give specific recommendations on the next diagnostic steps. They were asked to choose one or more of the following options: antibody testing, assessment of diaminoxydase, nutritional diary, elimination diet, H_2_ breathing test, histamine testing, provocation test, and test of urine and feces. To pass, students needed to mark one or more of the following options: provocation test, nutritional diary, or elimination diet, as these were the diagnostic factors recommended by a clinical expert.

### Data Analysis

Statistical analysis was performed using SPSS 25 (IBM Corp, Armonk, New York). Mean values, associated SDs, frequencies, and percentages of relevant factors such as age and gender were calculated. To test possible relationships among the variables, Chi-squared tests were used. Analysis of variance (ANOVAs) were conducted to compare differences between mean values. In addition, the Pearson correlations were calculated. Beforehand, distribution of data was tested by the Kolmogorov-Smirnov test. *P* values<.05 were reported as significant.

## Results

### Sample

There were 140 students who took part in the survey, and the response rate was 83.3% (140 /168). The average age was 24.36 (SD 2.84) years. Females constituted 61.7% (85) of the participating students. There were 50 students randomly assigned to Google, 46 to Medisuch, and 44 to the free choice group (*χ*^2^_278_=280.0, *P*=.46). All participants completed the accompanying worksheet.

There were no significant differences between the groups with regards to age (*F*_2,135_=5.04, *P*=.008), gender (*χ*^2^_4_=4.5, *P*=.34), and previous formal medical or information technology (IT) training (*χ*^2^_2_=1.5, *P*=.23). Smartphones were owned by 78.6% (110) of the students; only 2.1% (3) had a smartwatch; 46.4% (65) had a tablet; and 72.1% (111) had a laptop. The vast majority (131, 93.6%) reported spending from 1 to 6 hours online daily.

### Video Recording

Videos were successfully recorded from 125 students (44 from the Google group, 45 from the Medisuch group, and 36 from the free choice group). The remaining 15 recordings were lost due to technical reasons such as the recording or storing not working. The following data are based on these video recordings except those on diagnostic recommendation, which were based on the worksheet.

### Information-Seeking Behavior

There was no significant difference between groups regarding the total number of webpages students considered before making their diagnostic choice. The same result was shown for the number of pages regarded as useful. However, students of the free choice group (mean 0.88, SD 0.79) reported significantly fewer pages as recommendable to patients than the other two groups (*F*_2,133_=5.04, *P*=.008; M_Google_ 1.55, SD 0.91; M_Medisuch_ 1.52, SD 1.53).

**Figure 2:**
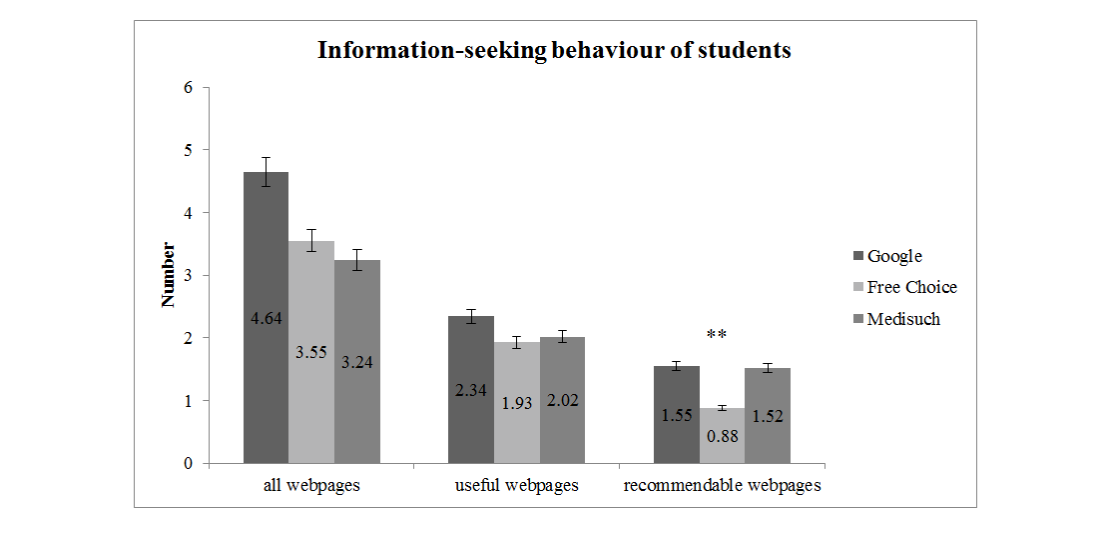
Information-seeking behavior of students regarding the total number of webpages.

Information seeking-behavior of students regarding the total number of webpages, the number of pages considered useful, and the number of pages considered recommendable for each group, as well as the means and SDs of the webpages are shown in Figure 2. Students in the free choice group opened significantly fewer recommendable pages (*F*_2,133_=5.04, *P*=.008).

**Figure 3:**
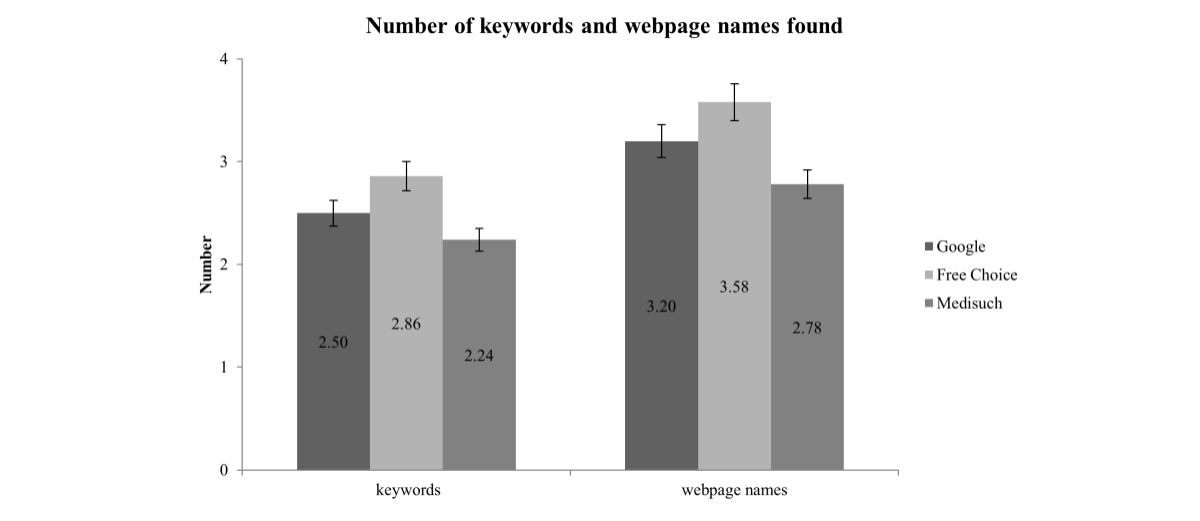
Number of keywords and webpage names found by students for each group.

There were no significant differences between groups in regards to the number of keywords entered in the search field or the number of webpages accessed (Figure 3).

There was no significant difference regarding the amount of keywords and webpage names found by students for each group. Means and the corresponding SDs are shown in Figure 3.

There was a highly significant difference between groups in whether or not the students entered specific medical terminology in the search engine (*χ*^2^_4_=16.6, *P*=.005). The majority of students in the Google and free choice groups entered either specific medical webpages such as DocCheck or specific medical terminology such as “evidence-based” into the search engine to specify their search; however, in the Medisuch group, more than one-third did not. Of the 44 students in the Google group, 38 (86%) used specific medical terminology for their search. In the free choice group 35 out of 36 (97%) students used specific medical terminology. In the Medisuch group 29 students out of 45 (64%) used specific medical terminology.

### Quality of Webpages

In total, 53 different webpages were accessed by the whole sample. Quality ratings showed that 34 pages fulfilled the criteria of a qualified webpage. Interrater-correlation after Cohen *d* was *r*=.73.

There were significantly high Pearson correlations between the number of webpages and the number of reliable webpages for all three groups (Google: *r*=.895; free group: *r*=.912; Medisuch: *r*=.860; all *P*<.001).

**Figure 4:**
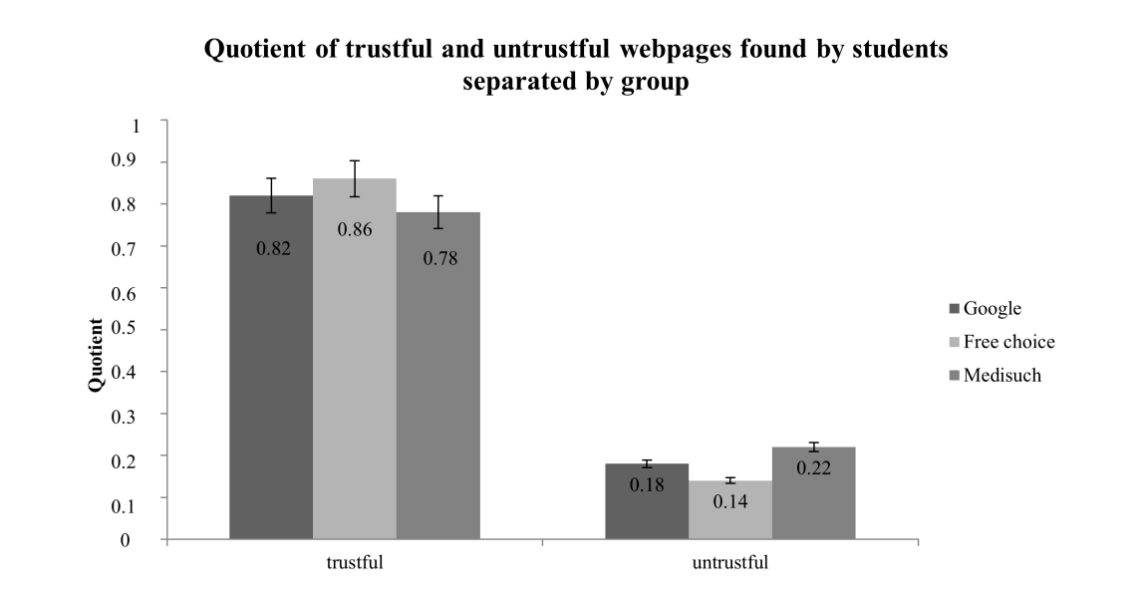
Quotient of trustworthy or untrustworthy webpages found by students separated by group.

There were no significant differences in the frequencies of trustworthy webpages found among the three groups with *χ*^2^_14_=16.45, *P*=.29 The webpage of German national treatment guidelines was used by students of all three groups (Google: 15.9%; free choice: 33.3%; and Medisuch: 51.1%). With regard to the quotient of reliable webpages and all webpages found by students, again, no significant difference was shown (F_2,121_=1.68, *P*=.19) between the groups. The mean quotients of the single groups were high with Google: 0.82 (SD 0.20); free group: 0.86 (SD 0.22); and Medisuch: 0.77 (SD 0.23) (Figure 4).

Figure 4 presents the means (and corresponding SDs) of the quotients of trustworthy or untrustworthy pages by the total number of pages found, which are separated by each group. There were no significant differences for trustworthy and untrustworthy sources.

### Diagnostic Recommendation

Independent of the search group, approximately 60% of students identified nutrition diary as a correct diagnostic step (Table 1). With regard to the provocation test, almost half the students found this procedure to be correct. The Medisuch student group reported the elimination diet as a correct diagnostic step for histamine intolerance more often than the Google or free choice groups. However, they suggested the wrong answer “antibody detection” significantly more often than the other groups.

**Table 1:** Diagnostic decision after online search.

Diagnostic step (correct or wrong)		Google (N=50)	Free choice (N=44)	Medisuch (N=46)	Chi-square (*df*)
**Antibody detection (wrong)**					
	yes	2 (4%)	1 (2%)	7 (15%)	6.84 (2)*, P*=.03
	no	48 (96%)	43 (98%)	39 (85%)	
**Histamine testing (wrong)**					
	yes	19 (38%)	15 (34%)	13 (28%)	1.03 (2),*P*=.60
	no	31 (62%)	29 (66%)	33 (72%)	
**Assessment of diaminoxydase (wrong)**					
	yes	29 (58%)	23 (52%)	18 (39%)	3.55 (2),*P*=.17
	no	21 (42%)	21 (48%)	28 (61%)	
**H_2_ breathing test (wrong)**					
	yes	0 (0%)	0 (0%)	0 (0%)	N/A^a^
	no	50 (100%)	44 (100%)	46 (100%)	
**Test of urine and feces (wrong)**					
	yes	7 (14%)	4 (9%)	7 (15 %)	0.84 (2),*P*=.66
	no	43 (86%)	40 (91%)	39 (85%)	
**Nutrition diary (correct)**					
	yes	30 (60%)	27 (61%)	28 (61%)	0.02 (2),*P*=.99
	no	20 (40%)	17 (39%)	18 (39%)	
**Elimination diet (correct)**					
	yes	26 (52%)	19 (43%)	33 (72%)	7.87 (2),*P*=.02
	no	24 (48%)	25 (57%)	13 (28%)	
**Provocation test (correct)**					
	yes	25 (50%)	21 (48%)	22 (48%)	0.06 (2),*P*=.97
	no	25 (50%)	23 (52%)	24 (52%)	

^a^Not applicable.

## Discussion

In this explanatory study, we examined medical students’ information-seeking behavior when assigned to use a generic search engine or a search engine of high quality. The hypothesis that students of the qualitatively better search engine would find the correct diagnostic steps for histamine intolerance more often was not fully supported. The Medisuch group significantly identified the elimination diet as a correct diagnostic step more frequently; however, this was not the case for the nutrition diary or the provocation test. The students of the Google and free choice groups reported the antibody detection as wrong more often than the Medisuch group. Furthermore, students of all three groups identified reliable webpages, which indicates that they do have internet skills that allow them to identify reliable information online. They were able to search for information online without navigation issues by entering specific webpages (like Flexicon) or medical terminology (diagnostic steps) in order to find reliable results [21]. Thus, students have successfully avoided the problem of standard search engine strategies, which often produce a multitude of results that users are forced to scroll through and sort the results [22]. This may be the reason why we did not find any differences between the groups using different search strategies.

A general question arises when viewing these results: Is entering specific terms or webpages into a generic search engine part of “digital literacy”? Many students searched at DocCheck [15], a webpage that is known for its trustworthy medical information. Students know that this webpage is a reliable source for information, as they use it for their medical studies [21,23,24]. Based on these results, medical students were able to comprehend the information found online and develop an understanding of how to find and evaluate it, which could be interpreted as information literacy [3,25]. Furthermore, medical students showed technical use competency by including specific terms in the search strategies [3,26], which previous studies have described as digital nativity [27,28]. Thus, information literacy and digital nativity were shown by the medical students involved in this study, which are determinants of online information search strategies. This point presents an argument for the medical students having digital literacy [3].

Finding health information online still includes the risk of being misinformed by unreliable information or information providers, which ultimately affects patients [29,30]. Consequently, medical students as future health professionals should have appropriate internet skills and use them to help patients find trustworthy information online. Additionally, online technologies for health information should be implemented in the medical curriculum [1].

One limitation of this study might be that search results and choices of relevant information is only one step out of many. It might be relevant to both track students’ search behavior and assess their cognitive processes preceding the search, such as defining the problem, choosing a certain source of information, or formulating the search strategies [30]. Additionally, in their feedback, students in this study reported that 10 minutes for conducting an information search seemed too short to gather sufficient information. However, as time in daily practice is limited, we consider this a realistic time frame to look for needed information. Furthermore, we need to consider that the number of reliable webpages could differ if other search engines or different topics were used. Generally, it can be argued that the free Web might not offer the same reliable information like customized webpages do (eg, UpToDate or AMBOSS). Future studies, thus, should focus on the patients’ online search strategies and use a similar setting to get more insights into general digital health literacy.

This study showed that medical students are able to search and find relevant medical information online regardless of the search engine used, and thus, this study confirms previous findings of medical students having internet skills in a professional sense. Future studies could focus on how to best integrate these internet skills into the medical curriculum. Furthermore, it needs to be determined if the online behavior of the students involved can be considered proper digital literacy. The next step could also focus on patients and their online search strategies.

## Abbreviations

ANOVA: analysis of variance
HONcode: Health On the Net Foundation Code of Conduct
IT: information technology.
