# Correction: Undergraduate Medical Students’ Search for Health Information Online: Explanatory Cross-Sectional Study

**DOI:** 10.2196/23253

**Published:** 2020-08-11

**Authors:** Teresa Loda, Rebecca Erschens, Florian Junne, Andreas Stengel, Stephan Zipfel, Anne Herrmann-Werner

**Affiliations:** 1 Medical Department VI/Psychosomatic Medicine and Psychotherapy University Hospital Tuebingen Tuebingen Germany; 2 Charité Center for Internal Medicine and Dermatology Department for Psychosomatic Medicine Charité-Universitätsmedizin Berlin Berlin Germany; 3 Corporate Member of Freie Universität Berlin Humboldt-Universität zu Berlin and Berlin Institute of Health Berlin Germany; 4 Deanery of Students’ Affairs Faculty of Medicine University Hospital Tuebingen Tuebingen Germany

In “Undergraduate Medical Students’ Search for Health Information Online: Explanatory Cross-Sectional Study” (JMIR Med Inform 2020;8(3):e16279) the authors noted errors in the presentation of the *P* values in the text of the Results section and in Table 1 of the published manuscript.

For the effected text in the Results section, under the "Sample" subheading, the following sentence was revised from:

There were 50 students randomly assigned to Google, 46 to Medisuch, and 44 to the free choice group (χ^2^_278_=280.0 *,P* P=).

To:

There were 50 students randomly assigned to Google, 46 to Medisuch, and 44 to the free choice group (χ^2^_278_=280.0,*P*=.46).

And this sentence was revised from:

There were no significant differences between the groups with regards to age (F_2,135_=5.04,*P*P=), gender (χ^2^_4_=4.5,*P*P=), and previous formal medical or information technology (IT) training (χ^2^_2_=1.5,*P*P=).

To:

There were no significant differences between the groups with regards to age (F_2,135_=5.04,*P*=.008), gender (χ^2^_4_=4.5,*P*=.34), and previous formal medical or information technology (IT) training (χ^2^_2_=1.5,*P*=.23).

Under the "Information-Seeking Behavior" subheading, the following sentence was revised from:

However, students of the free choice group (mean 0.88, SD 0.79) reported significantly fewer pages as recommendable to patients than the other two groups (F_2,133_=5.04,*P*P=; M_Google_ 1.55, SD 0.91; M_Medisuch_ 1.52, SD 1.53).

To:

However, students of the free choice group (mean 0.88, SD 0.79) reported significantly fewer pages as recommendable to patients than the other two groups (F_2,133_=5.04,*P*=.008; M_Google_ 1.55, SD 0.91; M_Medisuch_ 1.52, SD 1.53).

This sentence was revised from:

Students in the free choice group opened significantly fewer recommendable pages (F_2,133_=5.04,*P*P=).

To:

Students in the free choice group opened significantly fewer recommendable pages (F_2,133_=5.04,*P*=.008).

And this sentence was revised from:

There was a highly significant difference between groups in whether or not the students entered specific medical terminology in the search engine (χ^2^_4_=16.6,*P*P=).

To:

There was a highly significant difference between groups in whether or not the students entered specific medical terminology in the search engine (χ^2^_4_=16.6,*P*=.005).

Under the "Quality of Webpages" subheading, the following sentence was revised from:

There were significantly high Pearson correlations between the number of webpages and the number of reliable webpages for all three groups (Google: *r*=.895; free group: *r*=.912; Medisuch: *r*=.860; all*P* P<).

To:

There were significantly high Pearson correlations between the number of webpages and the number of reliable webpages for all three groups (Google: *r*=.895; free group: *r*=.912; Medisuch: *r*=.860; all *P*<.001).

This sentence was revised from:

There were no significant differences in the frequencies of trustworthy webpages found among the three groups with χ^2^_14_=16.45,*P*P=.

To:

There were no significant differences in the frequencies of trustworthy webpages found among the three groups with χ^2^_14_=16.45,*P*=.29.

And this sentence was revised from:

With regard to the quotient of reliable webpages and all webpages found by students, again, no significant difference was shown (F_2,121_=1.68,*P*P=) between the groups.

To:

With regard to the quotient of reliable webpages and all webpages found by students, again, no significant difference was shown (F_2,121_=1.68,*P*=.19) between the groups.

Additionally, for Table 1, the P values in the far right column, under the heading "Chi-square (*df*)" have also been revised.

The "Histamine testing (wrong)" row was revised from:

1.03 (2),*P*P=

To:

1.03 (2),*P*=.60

The "Assessment of diaminoxydase (wrong)" row was revised from:

3.55 (2),*P*P=

To:

3.55 (2),*P*=.17

The "Test of urine and feces (wrong)" row was from:

0.84 (2), *P*P=

To:

0.84 (2),*P*=.66

The "Nutrition diary (correct)" row was revised from:

0.02 (2), *P*P=

To:

0.02 (2),*P*=.99

The "Elimination diet (correct)" row was revised from:

7.87 (2), *P*P=

To:

7.87 (2),*P*=.02

The "Provocation test (correct)" row was revised from:

0.06 (2), *P*P=

To:

0.06 (2),*P*=.97

The correction will appear in the online version of the paper on the JMIR Publications website on August 11, 2020, together with the publication of this correction notice. Because this was made after submission to PubMed, PubMed Central, and other full-text repositories, the corrected article has also been resubmitted to those repositories.

